# Illicit Cannabis Use to Self-Treat Chronic Health Conditions in the United Kingdom: Cross-Sectional Study

**DOI:** 10.2196/57595

**Published:** 2024-08-14

**Authors:** Simon Erridge, Lucy Troup, Mikael Hans Sodergren

**Affiliations:** 1 Department of Surgery & Cancer, Imperial College London, Academic Surgical Unit, 10th Floor QEQM, St Mary’s Hospital, South Wharf Road, London, W2 1NY, United Kingdom, 44 203312666; 2 Curaleaf Clinic, London, United Kingdom; 3 University of the West of Scotland, Scotland, United Kingdom

**Keywords:** cannabis, chronic pain, anxiety, multiple sclerosis, posttraumatic stress disorder, PTSD, fibromyalgia, misuse, cannabis misuse, cannabis use, self-treatment, chronic health condition, cross-sectional study, United Kingdom, illicit cannabis, adult, consumption, adults, survey, cannabis-based, medicinal products, cannabis-based medicinal products

## Abstract

**Background:**

In 2019, it was estimated that approximately 1.4 million adults in the United Kingdom purchased illicit cannabis to self-treat chronic physical and mental health conditions. This analysis was conducted following the rescheduling of cannabis-based medicinal products (CBMPs) in the United Kingdom but before the first specialist clinics had started treating patients.

**Objective:**

The aim of this study was to assess the prevalence of illicit cannabis consumption to treat a medically diagnosed condition following the introduction of specialist clinics that could prescribe legal CBMPs in the United Kingdom.

**Methods:**

Adults older than 18 years in the United Kingdom were invited to participate in a cross-sectional survey through YouGov between September 22 and 29, 2022. A series of questions were asked about respondents’ medical diagnoses, illicit cannabis use, the cost of purchasing illicit cannabis per month, and basic demographics. The responding sample was weighted to generate a sample representative of the adult population of the United Kingdom. Modeling of population size was conducted based on an adult (18 years or older) population of 53,369,083 according to 2021 national census data.

**Results:**

There were 10,965 respondents to the questionnaire, to which weighting was applied. A total of 5700 (51.98%) respondents indicated that they were affected by a chronic health condition. The most reported condition was anxiety (n=1588, 14.48%). Of those enduring health conditions, 364 (6.38%) purchased illicit cannabis to self-treat health conditions. Based on survey responses, it was modeled that 1,770,627 (95% CI 1,073,791‐2,467,001) individuals consume illicit cannabis for health conditions across the United Kingdom. In the multivariable logistic regression, the following were associated with an increased likelihood of reporting illicit cannabis use for health reasons—chronic pain, fibromyalgia, posttraumatic stress disorder, multiple sclerosis, other mental health disorders, male sex, younger age, living in London, being unemployed or not working for other reasons, and working part-time (*P*<.05).

**Conclusions:**

This study highlights the scale of illicit cannabis use for health reasons in the United Kingdom and the potential barriers to accessing legally prescribed CBMPs. This is an important step in developing harm reduction policies to transition these individuals, where appropriate, to CBMPs. Such policies are particularly important considering the potential risks from harmful contaminants of illicit cannabis and self-treating a medical condition without clinical oversight. Moreover, it emphasizes the need for further funding of randomized controlled trials and the use of novel methodologies to determine the efficacy of CBMPs and their use in common chronic conditions.

## Introduction

In November 2018, the United Kingdom Home Office rescheduled cannabis-based medicinal products (CBMPs), allowing them to be initiated by consultant physicians on the General Medical Council’s specialist register for individuals who failed to achieve sufficient benefit from licensed therapies [1]. This was in response to the commentary provided by the then chief medical officer to suggest that there was conclusive evidence of the medicinal value of CBMPs [1]. At the end of 2022, it was estimated that 32,000 patients were now being prescribed CBMPs [2]. The most common conditions for which they are now prescribed include chronic pain, anxiety, and fibromyalgia [3]. However, there are still several barriers to eligible patients accessing CBMPs, including cost, perceived stigmatization, and a lack of high-quality randomized controlled trials (RCTs) [4-7]. Therefore, while there has been significant growth in the number of patients being prescribed CBMPs, this is surpassed by the most recent estimates of illicit cannabis use, including those who are using cannabis to self-treat diagnosed health conditions [8,9].

Cannabis is one of the most used drugs globally, and the United Kingdom is among the top 10 highest consumers of cannabis in Europe [8,10-13]. The incidence of cannabis use in the United Kingdom is continuing to rise in line with other countries [8,10-14]. In March 2013, the past-year prevalence of illicit cannabis use was 6.3% [8]. It has since risen to 7.6% [8]. While the perception of risk associated with cannabis is low and assessment of longitudinal registry data suggests CBMPs are largely well-tolerated [3,15-17], there are inherent personal and societal harms that may be associated with illicit cannabis use, even when intended for symptom management or control.

Illegal cannabis does not have to meet any regulatory standards to ensure consistency or absence of harmful contaminants. Several potentially pathogenic bacterial species have been identified in cannabis, including *Acinetobacter baumannii, Escherichia coli, Pseudomonas aeruginosa*, and *Clostridium botulinum* [18]. Moreover, several fungal species identified from dried cannabis flowers, including *Penicillium spp, Aspergillus spp,* and *Fusarium spp* commonly cause invasive infections in the immunocompromised [18]. The true incidence of adverse effects due to exposure to potential pathogens in illicit cannabis is not well characterized. Case reports indicate that the greatest risk is to immunocompromised individuals or those with underlying lung disease [18]. Yet, there are reports of invasive infections secondary to contaminated illicit cannabis in otherwise healthy individuals [19,20]. Moreover, the use of tainted fertilizers or phosphate-heavy fertilizers can lead to heavy metal contamination, specifically cadmium and arsenic, at levels exceeding those that are considered safe [18]. Inappropriate use of pesticides may also expose individuals to harmful compounds, including carcinogens [21].

There are other risks associated with illicit cannabis use. Between 2010 and 2020, there were 162,000 convictions in English and Welsh courts where drug possession was the most significant offense [22]. Overpolicing of drug possession disproportionately affects Black communities [22]. Beyond harm to the individual, illicit drug markets, including cannabis, actively contribute to the sustenance of organized crime groups and their exploitation of vulnerable individuals, including women, children, and refugees [23-26].

There is a paucity of high-quality RCTs to inform the evidence base on CBMPs [27,28]. Consequently, while there is promising evidence of its medicinal effects, this is currently insufficient to recommend their use on a population basis, except for a few specific indications for which there are licensed CBMPs [28,29]. There is, therefore, limited access to CBMPs in the United Kingdom. A cross-sectional, nationally representative survey on the prevalence of self-treating health conditions with illicit cannabis was conducted in October 2019 [9]. It estimated that 1.4 million individuals were consuming illicit cannabis for health reasons [9]. While that analysis was conducted following the rescheduling of CBMPs, it was completed before the first clinic meeting regulatory standards, Curaleaf Clinic (formerly known as Sapphire Medical Clinics), began seeing patients [30]. Consequently, the impact of rescheduling of CBMPs will not be incorporated in that study’s findings. Moreover, the report by Couch [9] has not undergone peer review. This study, therefore, primarily aimed to assess the prevalence of cannabis use for health conditions, as the effect of access to CBMPs on illicit cannabis consumption is unknown. This study also aimed to assess which demographic factors are associated with an increased likelihood of consuming illicit cannabis for health reasons.

## Methods

### Study Design

A cross-sectional survey was administered to adults (18 years or older) residing in the United Kingdom between September 22 and 29, 2022. The survey was distributed to a nationally representative sample by YouGov (YouGov PLC). Participants were recruited using active sampling by YouGov from a panel of more than 800,000 individuals [31]. This method was used to generate a nationally representative sample of UK adults.

### Ethical Considerations

Participants provide YouGov with consent to be contacted via email and participate in questionnaires. YouGov is a member of the British Polling Council and the European Society for Opinion and Marketing Research. It is also registered with the Information Commissioner. Participants are rewarded for taking part in surveys by receiving points, which can be converted to financial compensation. As all data were anonymized, there was no need for participants to provide further consent, beyond the implicit consent by completing the survey. The survey was developed by the study authors and reviewed by YouGov to ensure compliance with their Global Code of Ethics. The questionnaire was distributed on behalf of the authors by YouGov. Access to anonymized data was provided to the authors following the completion of the study period. The University of the West of Scotland School of Education and Social Sciences Ethics Committee provided a retrospective exemption for this study (# 2024-21236-17820).

### Study Overview

The survey was developed using the questionnaire developed previously in a report by Couch [9]. This was to allow for a direct comparison of prevalence between each analysis. Changes were made to the questionnaire to account for the differences between October 2019 and September 2022 in access to specialist medical cannabis clinics who could prescribe CBMPs. Questions were delivered in series with branching logic applied between questions 1 and 2, removing respondents who reported they did not have any diagnosed health conditions. Branching logic was also applied between question 2 and the rest of the survey, removing participants who did not use illicit cannabis for their health condition or were not prepared to disclose their use. The questions are detailed in full in Table 1.

**Table 1.** Questionnaire and available responses administered to a nationally representative sample via YouGov to adults (18 years or older) residing in the United Kingdom between September 22 and 29, 2022.

Question	Answers
For the following questions, please remember your answers will always be treated anonymously and will never be analyzed individually...Which, if any, of the following health conditions have you been diagnosed with officially by a medical professional (eg, a doctor or nurse) as currently having? (Please select all that apply)	Chronic pain Anxiety disorder Fibromyalgia Post-traumatic stress disorder (PTSD) Multiple sclerosis (MS) Other mental health condition Other physical condition Other, not described Not applicable—I have not been diagnosed with any health condition
For the following questions, when answering the question, please think only about the plant product cannabis (also known as marijuana) that you are not able to obtain from a specialist doctor.Please do not include cannabis derived products (like cannabidiol or CBD) that you can legally obtain on the high street or internet, or those available on prescription from a specialist doctor...	N/A^a^
As a reminder, please remember your answers will always be treated anonymously and will never be analyzed individually. Thinking only about any use of cannabis to specifically manage your condition(s) mentioned in the previous question, or to treat any symptoms or side effects brought on by your prescribed treatment, please do not include using cannabis recreationally or for any other purpose...Do you currently use cannabis to help manage or treat any symptoms of your condition(s) or side effects of its treatment?	Yes, I currently use cannabis to help manage or treat symptoms of my condition(s) or side effects brought on by treatment No, I do not currently use Don’t know Prefer not to say
Approximately, how much money would you say you personally spend on cannabis to manage or treat some symptoms or side effects brought on by the treatment of your condition(s), in an average month?	£1^b^ to £99 £100 to £199 £200 to £299 £300 to £399 £400 or above Don’t know Not applicable—I do not spend any money Prefer not to say
As a reminder, your answers will always be treated anonymously and will never be analyzed individually. Which, if any, of the following are reasons why you obtain cannabis for your condition(s in the way you currently do? (Please select all that apply. If any of your answers don’t appear in the list below, please type them in the “Other” box)	I was not aware that it is available legally to manage or treat some physical or mental health conditions I presume obtaining it legally would be very difficult I presume obtaining it legally would be very expensive I want to manage or treat my condition quickly Other Don’t know Prefer not to say
For the following question, by “medical cannabis,” we mean any cannabis-based medicine prescribed by a GP^c^ or specialist. Thinking about speaking to a GP or specialist about medical cannabis being used to manage or treat your current physical or mental health condition(s)...Which one, if any of the following statements best applies to you? (If you have spoken to a GP or specialist about medical cannabis more than once, please think about the most recent time)	We have discussed it in detail and explored it or are exploring it further We have discussed it in detail and decided against it My GP or specialist mentioned it in passing I mentioned it but my GP or specialist advised against it I mentioned it to my GP or specialist but they knew nothing about it We have never discussed it None of these Don’t know or can’t recall Prefer not to say

a N/A: not applicable.

b At the time the study was conducted the exchange rate was £1 GBP to US $1.08.

c GP: general practitioner.

In addition, the demographic data that were recorded are age, gender, geographical region, government region, working status, marital status, number of children in the household, parent or guardian status, and use of social media or messaging services in the last month. The social grade was also recorded as either middle class or working class or nonworking individuals as defined by the National Readership Survey social grade classifications [32]. At the time the study was conducted the exchange rate was £1 GBP to US $1.08.

### Data Collection

In accordance with YouGov active sampling methodology, participants were selected at random from the base sample of over 800,000 individuals. These emails are generic and do not alert the participant to the subject matter prior to engaging with the questions. The invitation link was active for this survey throughout the data collection period. This methodology, when used in conjunction with proportional weighting to a population-matched sample has been shown to create a sample that is representative of the UK adult population [31]. This has been demonstrated in predicting public opinion on political and social issues [31].

### Statistical Analysis

The responses to the questionnaire underwent proportional weighting to ensure they were representative of the target population, adult residents of the United Kingdom. The respondent number was subsequently rounded to the nearest integer. All other data are presented in 2 decimal places. The prevalence of each condition and reported illicit cannabis use was calculated and presented with 95% CIs. All other responses are reported as frequencies. Modeling of population size was conducted based on an adult (18 years or older) population of 53,369,083 according to 2021 national census data. A univariable logistic regression was conducted to assess the association between the condition and demographic variables and the likelihood of reporting illicit cannabis use for health reasons. All variables with a statistically significant outcome were taken forward into a multivariable logistic regression analysis. They were reported as odds ratios (ORs) and 95% CIs. *P* values <.05 were considered statistically significant. All statistical analysis was conducted using R (version 4.3.1; R Core Team). Figures were created using the ggplot2 package.

## Results

### Prevalence of Health Conditions

**Figure 1.**
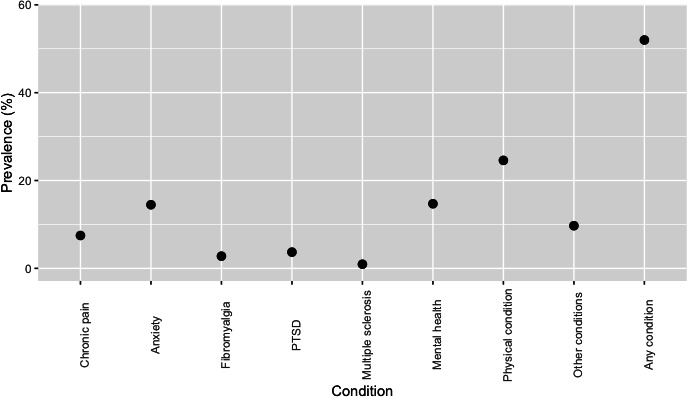
Prevalence (%) of medical conditions diagnosed by a health care professional reported by respondents to a nationally representative survey. Mental health means other mental health condition; other conditions means other, not described; and physical condition means other physical condition. PTSD: posttraumatic stress disorder.

There were 10,965 respondents to the questionnaire. After weighting was applied, 5700 (51.98%) participants reported experiencing any diagnosed health conditions (Figure 1). The number of UK adults estimated to be affected by a diagnosed health condition was subsequently modeled as 27,741,361 (95% CI 27,242,290‐28,240,433; Multimedia Appendix 1). The most reported diagnoses were the other physical condition (24.58%, 95% CI 23.77%‐25.39%) and other mental health condition (14.71%, 95% CI 14.05%‐15.37%) groups. The most common specifically identified conditions were anxiety (14.48%, 95% CI 13.82%‐15.14%) and chronic pain (7.48%, 95% CI 6.99%‐7.98%).

A total of 3072 (53.90%) participants with health conditions were female. The most common age category was 55 years and older (n=2656; 46.59%). The full demographics of the population with health conditions, with additional stratification according to individual condition is detailed in Multimedia Appendix 2.

### Reported Use of Illicit Cannabis for Health Conditions

**Figure 2.**
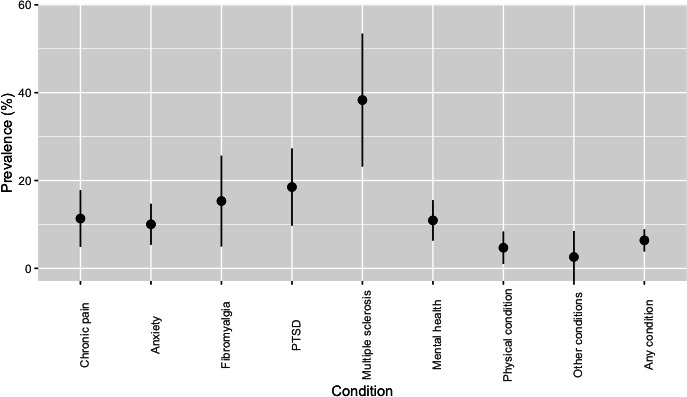
Prevalence (%) of illicit cannabis consumption reported by respondents to a nationally representative survey for medical conditions diagnosed by a health care professional. Mental health means other mental health condition; other conditions means other, not described; and physical condition means other physical condition. PTSD: posttraumatic stress disorder.

Of the 5700 participants who declared having 1 or more health conditions captured by this survey, 364 (6.38%) reported using illicit cannabis specifically to manage the declared condition (Figure 2). The total population of UK adults who use illicit cannabis to manage health conditions was estimated as 1,770,627 (95% CI 1,073,791‐2,467,001; Table 2). The condition with the highest proportional prevalence of illicit cannabis use was multiple sclerosis (38.31%, 95% CI 23.19%‐53.43%). The specific condition with the largest estimated population of self-treating with illicit cannabis in the United Kingdom was anxiety (n=775,782, 95% CI 415,243‐1,136,107).

**Table 2.** Illicit cannabis consumption reported by survey respondents to self-treat medical conditions diagnosed by a health care professional.

Condition	Respondents, n	Prevalence (%; 95% CI)	UK population estimate (95% CI)
Chronic pain	93	11.35 (4.91 to 17.79)	453,255 (195,947 to 710,430)
Anxiety	159	10.04 (5.37 to 14.70)	775,782 (415,243 to 1,136,107)
Fibromyalgia	47	15.32 (5.00 to 25.64)	227,752 (74,355 to 381,077)
PTSD^a^	75	18.51 (9.73 to 27.30)	365,421 (191,994 to 538,747)
Multiple sclerosis	40	38.31 (23.19 to 53.43)	193,336 (117,038 to 269,583)
Other mental health condition	176	10.93 (6.32 to 15.53)	857,760 (496,165 to 1,219,127)
Other physical condition	127	4.70 (1.01 to 8.38)	616,088 (132,613 to 1,099,357)
Other, not described	28	2.59 (−3.34 to 8.52)	134,184 (−173,035 to 441,321)
Any condition	364	6.3 (3.87 to 8.89)	1,770,627 (1,073,791 to 2,467,001)

a PTSD: posttraumatic stress disorder.

A total of 201 (55.20%) participants who consumed illicit cannabis for this reason were male. The most common age category was 18‐24 years (n=106, 29.15%). A total of 175 (44.07%) participants were in full-time or part-time employment. The full demographics of individuals who consume illicit cannabis for health reasons are contained within Multimedia Appendix 3.

Univariable logistic regression analysis identified that the presence of each condition, gender, age category, social grade, geographic region, reemployment status, marital status, and the number of children in the household were associated with the likelihood of consuming illicit cannabis for a diagnosed health condition (*P*<.05; Table 3).

**Table 3.** Univariable logistic regression analysis of the relationship between independent variables and the likelihood of consuming illicit cannabis to self-manage a diagnosed health condition.

		Values, n	Odds ratio (95% CI)	*P* value
**Chronic pain**				
	No	4921	Ref^a^	N/A^b^
	Yes	839	2.17 (1.67‐2.78)	<.001
**Anxiety**				
	No	4239	Ref	N/A
	Yes	1521	2.12 (1.71‐2.63)	<.001
**Fibromyalgia**				
	No	5461	Ref	N/A
	Yes	299	2.88 (2.07‐4.01)	<.001
**PTSD** ^**c**^				
	No	5372	Ref	N/A
	Yes	388	3.92 (2.97‐5.17)	<.001
**Multiple sclerosis**				
	No	5666	Ref	N/A
	Yes	94	10.04 (6.56‐15.16)	<.001
**Other mental health condition**				
	No	4205	Ref	N/A
	Yes	1555	2.53 (2.04‐3.14)	<.001
**Other physical condition**				
	No	2928	Ref	N/A
	Yes	2832	0.57 (0.46‐0.72)	<.001
**Other condition, not described**				
	No	4642	Ref	N/A
	Yes	1118	0.34 (0.23‐0.50)	<.001
**Sex**				
	Female	3164	Ref	N/A
	Male	2596	1.50 (1.21‐1.85)	<.001
**Age (years)**				
	18‐24	405	Ref	N/A
	25‐34	701	0.60 (0.44‐0.81)	<.001
	35‐44	883	0.46 (0.33‐0.62)	<.001
	45‐54	850	0.25 (0.17‐0.36)	<.001
	≥55	2921	0.07 (0.05‐0.10)	<.001
**Social grade**				
	ABC1^d^	3336	Ref	N/A
	C2DE^e^	2424	1.32 (1.07‐1.63)	.01
**Region**				
	London	651	Ref	N/A
	East Midlands	416	0.42 (0.25‐0.70)	<.001
	East of England	498	0.42 (0.26‐0.68)	<.001
	North East	237	0.57 (0.32‐1.01)	.05
	North West	629	0.63 (0.43‐0.93)	.02
	Northern Ireland	157	1.04 (0.60‐1.81)	.89
	Scotland	513	0.52 (0.33‐0.81)	.004
	South East	777	0.44 (0.29‐0.66)	<.001
	South West	574	0.43 (0.28‐0.68)	<.001
	Wales	297	0.70 (0.43‐1.14)	.15
	West Midlands	494	0.43 (0.27‐0.70)	<.001
	Yorkshire and the Humber	517	0.53 (0.35‐0.83)	.005
**Employment status**				
	Working full time	1963	Ref	N/A
	Full-time student	189	2.963 (1.992‐4.408)	<.001
	Not working other	681	2.089 (1.542‐2.828)	<.001
	Retired	1887	0.248 (0.159‐0.386)	<.001
	Unemployed	249	3.440 (2.394‐4.943)	<.001
	Working part time	791	1.454 (1.056‐2.000)	.02
**Marital status**				
	Never married	1477	Ref	N/A
	Living as married	710	0.54 (0.39‐0.76)	<.001
	Married or civil partnership	2680	0.29 (0.23‐0.38)	<.001
	Separated or divorced	598	0.43 (0.29‐0.64)	<.001
	Widowed	295	0.19 (0.09‐0.43)	<.001
**Children in household**				
	No children	4541	Ref	N/A
	1 child	572	1.53 (1.11‐2.11)	.01
	2 children	459	1.57 (1.10‐2.23)	.01
	≥3 children	188	4.11 (2.84‐5.93)	<.001

a Ref: reference.

b N/A: not applicable.

c PTSD: posttraumatic stress disorder.

d ABC1: middle class.

e C2DE: working class or not working.

Multivariable logistic regression analysis was conducted including variables that were statistically significant on univariable analysis. Chronic pain, fibromyalgia, posttraumatic stress disorder (PTSD), multiple sclerosis, other mental health conditions, gender, age category, geographic region, employment status, and number of children in the household were identified as having an association with consuming illicit cannabis for a diagnosed health condition (*P*<.05; Table 4).

**Table 4.** Multivariable logistic regression analysis of the relationship between independent variables and the likelihood of consuming illicit cannabis to self-manage a diagnosed health condition.

		Values, n	Odds ratio (95% CI)	*P* value
**Chronic pain**				
	No	4921	Ref^a^	N/A^b^
	Yes	839	2.01 (1.49‐2.71)	<.001
**Anxiety**				
	No	4239	Ref	N/A
	Yes	1521	1.00 (0.77‐1.29)	.99
**Fibromyalgia**				
	No	5461	Ref.	N/A
	Yes	299	1.77 (1.19‐2.65)	.005
**PTSD** ^c^				
	No	5372	Ref	N/A
	Yes	388	2.43 (1.78‐3.32)	<.001
**Multiple sclerosis**				
	No	5666	Ref	N/A
	Yes	94	7.47 (4.58‐12.20)	<.001
**Other mental health condition**				
	No	4205	Ref	N/A
	Yes	1555	1.41 (1.10‐1.81)	.008
**Other physical condition**				
	No	2928	Ref	N/A
	Yes	2832	0.92 (0.71‐1.20)	.53
**Other condition, not described**				
	No	4642	Ref	N/A
	Yes	1118	0.68 (0.44‐1.05)	.08
**Sex**				
	Female	3164	Ref	N/A
	Male	2596	1.86 (1.46‐2.37)	<.001
**Age (years)**				
	18‐24	405	Ref	N/A
	25‐34	701	0.72 (0.50‐1.03)	.07
	35‐44	883	0.52 (0.36‐0.76)	<.001
	45‐54	850	0.27 (0.17‐0.42)	<.001
	≥55	2921	0.10 (0.06‐0.17)	<.001
**Social grade**				
	ABC1^d^	3336	Ref	N/A
	C2DE^e^	2424	1.16 (0.90‐1.50)	.25
**Region**				
	London	651	Ref	N/A
	East Midlands	416	0.48 (0.28‐0.85)	.01
	East of England	498	0.48 (0.29‐0.80)	.005
	North East	237	0.57 (0.30‐1.06)	.08
	North West	629	0.75 (0.48‐1.15)	.19
	Northern Ireland	157	1.14 (0.62‐2.10)	.67
	Scotland	513	0.51 (0.31‐0.84)	.008
	South East	777	0.50 (0.29‐0.70)	<.001
	South West	574	0.50 (0.31‐0.83)	.007
	Wales	297	0.72 (0.42‐1.25)	.25
	West Midlands	494	0.43 (0.26‐0.72)	.001
	Yorkshire and the Humber	517	0.53 (0.33‐0.87)	.01
**Employment status**				
	Working full time	1963	Ref	N/A
	Full-time student	189	1.18 (0.74‐1.91)	.49
	Not working other	681	1.71 (1.19‐2.45)	.003
	Retired	1887	1.08 (0.61‐1.93)	.79
	Unemployed	249	2.26 (1.49‐3.43)	<.001
	Working part time	791	1.82 (1.27‐2.61)	.001
**Marital status**				
	Never married	1477	Ref	N/A
	Living as married	710	0.80 (0.56‐1.16)	.24
	Married or civil partnership	2680	0.84 0.60‐1.16)	.29
	Separated or divorced	598	1.32 (0.83‐2.10)	.25
	Widowed	295	0.97 (0.38‐2.47)	.95
**Children in household**				
	No children	4541	Ref	N/A
	1 child	572	0.89 (0.62‐1.27)	.52
	2 children	459	0.88 (0.58‐1.34)	.57
	≥3 children	188	1.61 (1.04‐2.48)	.03

a Ref: reference.

b N/A: not applicable.

c PTSD: posttraumatic stress disorder.

d ABC1: middle class.

e C2DE: working class or not working.

### Monthly Cost of Illicit Cannabis

**Figure 3.**
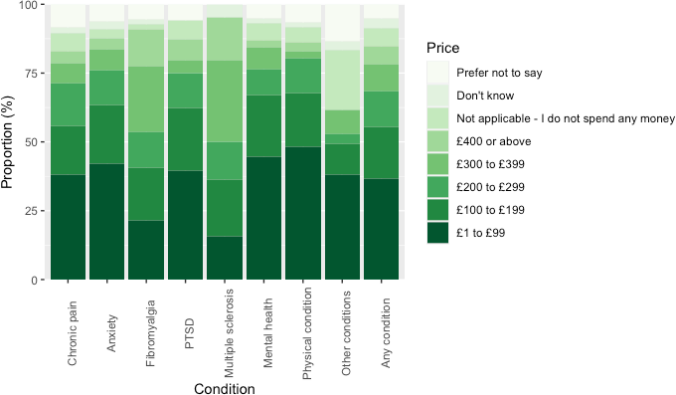
Proportional spend on illicit cannabis for a diagnosed health condition by respondents reported as a proportion of patients declaring illicit cannabis use to self-treat health conditions. At the time the study was conducted the exchange rate was £1 GBP to US $1.08. Mental health means other mental health condition; other conditions means other, not described; and physical condition means other physical condition. PTSD: posttraumatic stress disorder.

The most common monthly cost category was £1 to £99 (n=134, 36.85%). However, 68 (18.69%), 47 (12.85%), 36 (9.88%), and 24 (6.59%) respondents spent reported costs of £100 to £199, £200 to £299, £300 to £399, or £400 or above, respectively (Figure 3 and Multimedia Appendix 4).

### Reasons for Consuming Cannabis Illicitly

**Figure 4.**
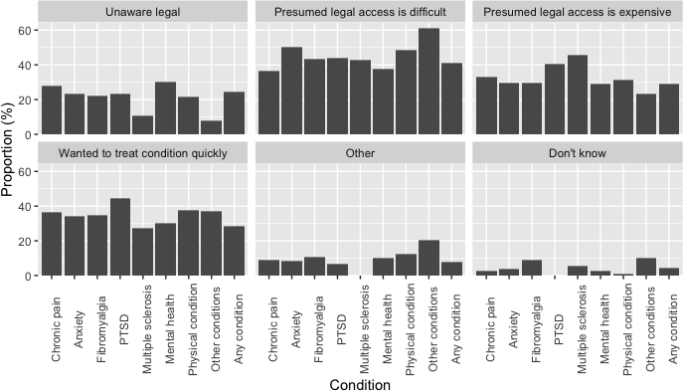
Reasons why respondents consumed illicit cannabis to self-treat their health conditions reported as a proportion of patients declaring illicit cannabis use to self-treat health conditions. Mental health means other mental health condition; other conditions means other, not described; and physical condition means other physical condition. NA: not applicable; PTSD: posttraumatic stress disorder.

On questioning as to why the participants chose to consume cannabis illicitly, the most common response was that they presumed legal access was very difficult (n=148, 40.75%) (Figure 4 and Multimedia Appendix 5). Participants could select more than 1 answer and other responses included that they presumed legal access was expensive (n=105, 28.87%), they wanted to treat their condition quickly (n=103, 28.35%), or that they were unaware it was legal (n=88, 24.15%).

### Discussion With Doctors

**Figure 5.**
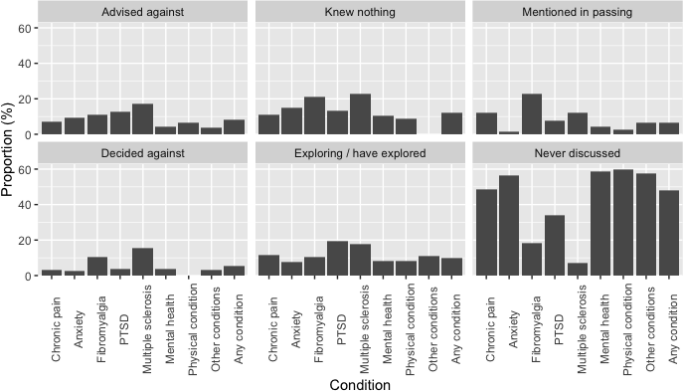
Outcome of discussions with general practitioner or specialist regarding cannabis-based medicinal products reported as a proportion of patients declaring illicit cannabis use to self-treat health conditions. Mental health means other mental health condition; other conditions means other, not described; and physical condition means other physical condition. PTSD: posttraumatic stress disorder.

When asked to consider their discussions with either a general practitioner (GP) or specialist doctor, 48.11% (n=175) said they had never discussed it (Figure 5 and Multimedia Appendix 6). A total of 11.86% (n=43) of respondents said they had discussed CBMPs with either a GP or specialist doctor, but they knew nothing about them. Considering those participants who had discussed it with their physician, 11.86% (n=43) said their doctor knew nothing about them, 9.71% (n=35) are exploring the option further or have explored it, 5.18% (n=19) decided against it in collaboration with their doctor, and 8.40% (n=31) were advised against CBMPs by their doctor.

## Discussion

This nationally representative survey study estimates that 6.38% of individuals with a diagnosed health condition consume cannabis illicitly as a component of self-treating that health condition. Using census data, this estimates that 1.77 million UK adults are using illicit cannabis for this reason. The health conditions with the strongest association for cannabis use on multivariate analysis were multiple sclerosis, chronic pain, and PTSD. The demographic factors with the strongest association with illicit cannabis use for health reasons included male sex, younger age categories, living in London, and being unemployed. The most commonly reported reason for using cannabis illicitly, rather than opting for legally prescribed CBMPs was due to presumed difficulties in accessing CBMPs. One in 4 illicit cannabis users were unaware that CBMPs had been rescheduled and could be legally prescribed in the United Kingdom. Moreover, almost half of the respondents who used illicit cannabis in this way had never discussed whether CBMPs may be an option for them.

The most striking finding from this study is that 1.77 million people were estimated to use illicit cannabis to treat their diagnosed health conditions based on the best available survey data. This is an increase from the only previous nationally representative study that has sought to quantify the population of UK adults who use illicit cannabis for this reason [9]. That report, which was conducted in 2019 but has not undergone peer review, before the introduction of specialist medical cannabis clinics, but after the rescheduling of CBMPs in the United Kingdom, estimated the figure to be 1.4 million [9]. While this previous study did not present the 95% CIs for this estimate it is important to note that the lower bound of the figure derived in this study is 1.07 million, which may, therefore, reflect that there has been no change. The difference could, therefore, simply represent natural variance in repeated surveying of similar populations. While this study used a very similar methodology to the study conducted in 2019, there are some key differences, which may also be reflected in the modeled population estimate. This study used terminology to capture individuals with any diagnosed health condition incorporating variables, such as other mental health condition; other physical condition; and other, not described. The 2019 study, in comparison used a longer list of specific diagnoses, but could not be exhaustive due to limitations of a survey study and did not use a catch-all term [9]. Consequently, the prevalence of any diagnosed health condition was 51.98% in this study, compared to 46.37% in the 2019 report [9]. Both figures are similar to the estimated prevalence derived from the UK sample of the European Health Interview Survey for 2019‐2020 (48.06%) [33]. The 95% CIs of estimated illicit cannabis use for health reasons overlap between 2019 and 2022, suggesting there was no change to the overall proportion of people who consume cannabis. This is supported by a study by Waldron et al [34] that found perception of risk toward both CBMPs, and recreational cannabis is unchanged despite the rescheduling of CBMPs.

Considering the potential health and societal harms that may be associated with illicit cannabis [18-21,23-26], irrespective of potential medicinal value, it is important to consider policy interventions that may facilitate the transition of patients from illicit cannabis to legal CBMPs with clinical oversight. Despite rescheduling, there may be many factors that mean participants continue to consume illicit cannabis. The study highlights a general lack of awareness of the rescheduling of CBMPs, with 1 in 4 participants being unaware of their legal status. Many participants also highlighted that they thought access to CBMPs may be difficult, expensive, or not appropriate to get timely treatment for their condition. Almost half of all individuals using illicit cannabis for self-treating their health condition had not talked about CBMPs with their GP or specialist. This may be reflective of the high levels of perceived stigma among patients using medical cannabis [4]. One in 5 individuals reported that their doctor had either advised against CBMPs or did not know enough about these medications. This is supported by data from the Primary Care Cannabis Network, which suggest 72% and 68% of GPs are concerned about the unlicensed nature of most CBMPs and lack of efficacy, respectively [35]. There may be supplementary barriers to accessing CBMPs that are not assessed in this study. Most care for individuals prescribed CBMPs is provided in the private sector [36,37]. The associated costs of this care may mean that it is not accessible to all. In addition, patients must meet national criteria for eligibility for CBMPs [38]. Therefore, patients who have not had a sufficient trial of licensed therapies will not be able to access CBMPs. Policy interventions, specifically targeted at overcoming these barriers to access may have positive implications with respect to harm reduction. Implementation of National Health Service (NHS) provisions to access CBMPs and care for individuals who report positive impacts on their diagnosed health conditions from illicit cannabis, and otherwise meet relevant eligibility criteria [38], may have positive effects on an individual and population basis. Ultimately, improved quality and quantity of clinical research will be required to truly address barriers to accessing CBMPs. At present, there is insufficient evidence to support national prescribing via the NHS [29]. Research with CBMPs that demonstrates cost-effectiveness in appropriate conditions will help reduce financial barriers, increase health care practitioner education, and help reduce stigma.

This study highlights differences between groups that may influence their likelihood of self-treating their health conditions with illicit cannabis. Multiple sclerosis had the strongest association with illicit cannabis in the multivariate logistic regression. Patients with multiple sclerosis were also more likely to report higher levels of expenditure on illicit cannabis. Multiple sclerosis was also the single condition that was most likely to be aware that CBMPs were available legally on prescription and to have discussed its use with a doctor. These findings may be secondary to awareness of nabiximols, a licensed CBMP for spasticity in adults with multiple sclerosis [29]. While this may serve to increase awareness of CBMPs as a treatment class, nabiximols are only available in restricted settings [29]. Chronic pain is the most common reason why CBMPs are prescribed in the United Kingdom and is the most common indication for symptomatic treatment in multiple sclerosis, but nabiximols are not available in this setting [39].

Observational or real-world evidence has played a crucial role in advancing the field of cannabis science in the absence of a sufficient number of high-quality RCTs. In the United Kingdom, for example, the efficacy of CBMPs in treating rare, treatment-resistant forms of epilepsy in select individuals was an important factor in the rescheduling of CBMPs [1]. As this study uncovers, 1.77 million people in the United Kingdom are estimated to consume illicit cannabis for health reasons. This is observational evidence of the potential therapeutic value of CBMPs, but insufficient to support wider access to CBMPs for individuals who are cannabis naïve. It does, however, support the need for further funding for RCTs of CBMPs in conditions such as chronic pain and anxiety, which are estimated in this study to affect 3.99 and 7.73 million UK adults, respectively. Considering the inherent challenges in conducting RCTs with CBMPs [40], novel approaches to incorporating and analyzing real-world evidence should also be considered. Large patient registries, such as the UK Medical Cannabis Registry, may be used in increasingly novel and innovative ways to further understand the clinical efficacy of CBMPs, beyond the preliminary data that have been published on chronic pain, anxiety, fibromyalgia, and PTSD so far [41-47].

Despite the usage of a sampling and weighting methodology to derive a nationally representative population, this study is subject to inherent limitations. Responses to the survey may be affected by a social desirability bias [48]. While all responses were anonymous, it is still well-known that participants in research are more likely to provide responses that are deemed acceptable. This may, therefore, lead to a reduction in declared illicit cannabis use for health reasons. YouGov uses an internet-based sampling methodology, which may inappropriately exclude individuals who cannot engage with digital technology. This may disproportionately affect certain members of society and, therefore, the representativeness of the survey [49]. While the weighting of the survey is adjusted to account for this sampling bias, there may still be characteristics of those who lack digital inclusivity that are unable to be accounted for by statistical weighting. The weighting of YouGov data also does not account for ethnicity or race, and therefore, information about this variable is not included in this analysis. Considering how overpolicing of cannabis possession disproportionately affects Black communities [22], further information on ethnicity would have been beneficial. Another limitation is the self-reporting of conditions. While efforts were made to word the first question appropriately to specifically ask about conditions diagnosed by an appropriately trained health care professional, without confirmation from a health care professional of the diagnostic accuracy it may lead to inappropriate recording of conditions. In addition, the study was limited to asking about 5 specific conditions, rather than having a more discrete list of other conditions that people may self-treat with illicit cannabis. Further conditions were not added due to cost constraints. This limits the additional analysis that can be assessed, such as the more comprehensive list published by Ware et al [50]. While this reduces the granularity of available data, by including terms to capture any other medical conditions, this is likely to improve the accuracy of the estimated total population of UK adults who use illicit cannabis for health reasons.

In conclusion, this study estimates that 1.77 million UK adults are consuming illicit cannabis for the purpose of managing their health conditions based on nationally representative survey data. This number has not materially changed since 2019 when it was estimated to be 1.4 million [9]. This is despite the introduction of specialist medical cannabis clinics that provide clinical care to an estimated 32,000 individuals in the United Kingdom [2]. To address the potential public health and societal problems this creates, despite any therapeutic value derived from illicit cannabis, it is important to prioritize policies that help reduce the barriers to accessing CBMPs. This is particularly important for the estimated 1.77 million UK adults who are consuming illicit cannabis for health reasons. Beyond this, it is important to prioritize funding and the adoption of novel research methodologies to establish the efficacy of CBMPs and the role they should play in the treatment of chronic health conditions for all individuals.

## Supplementary material

10.2196/57595 Multimedia Appendix 1 Medical conditions diagnosed by a health care professional reported by survey respondents.

10.2196/57595 Multimedia Appendix 2 Demographics of respondents with 1 or more medical conditions diagnosed by a health care professional.

10.2196/57595 Multimedia Appendix 3 Demographics of respondents who consume illicit cannabis to self-treat 1 or more medical conditions diagnosed by a health care professional.

10.2196/57595 Multimedia Appendix 4 Reported monthly expenditure on illicit cannabis for self-treating diagnosed health conditions.

10.2196/57595 Multimedia Appendix 5 Reasons why respondents consumed illicit cannabis to self-treat their health conditions.

10.2196/57595 Multimedia Appendix 6 Outcome of discussions with general practitioner or specialist regarding cannabis-based medicinal products.

## Abbreviations

CBMP: cannabis-based medicinal product
GP: general practitioner
NHS: National Health Service
OR: odds ratio
PTSD: posttraumatic stress disorder
RCT: randomized controlled trial
