# Supplementary material for: Illicit Cannabis Use to Self-Treat Chronic Health Conditions in the United Kingdom: Cross-Sectional Study
Source: JMIR Public Health Surveill. 2024 Aug 14;10:e57595. doi: 10.2196/57595 (PMC11337234; doi:10.2196/57595)
Supplement: Multimedia Appendix 1 [file publichealth-v10-e57595-s001.docx]

| Condition | Respondents (n) | Prevalence  (95% CI) | UK Population Estimate (95% CI) |
| --- | --- | --- | --- |
| Chronic Pain | 821 | 7.48%  (6.99-7.98%) | 3,994,285  (3,731,430-4,257,140) |
| Anxiety | 1,588 | 14.48%  (13.82-15.14%) | 7,728,028  (7,376,503-8,079,553) |
| Fibromyalgia | 305 | 2.78%  (2.48-3.09%) | 1,486,255  (1,321,892-1,650,617) |
| PTSD | 406 | 3.70%  (3.35-4.05%) | 1,974,048  (1,785,516-2,162,579) |
| Multiple Sclerosis | 104 | 0.95%  (0.76-1.13%) | 504,682  (408,003-601,362) |
| Other mental health condition | 1,613 | 14.71%  (14.05-15.37%) | 7,850,828  (7,496,998-8,204,658) |
| Other physical condition | 2,695 | 24.58%  (23.77-25.39%) | 13,117,843  (12,687,748-13,547,938) |
| Other, not described | 1,065 | 9.71%  (9.16-10.26%) | 5,182,179  (4,886,402-5,477,956) |
| Any Condition | 5,700 | 51.98%  (51.05-52.92%) | 27,741,361  (27,242,290-28,240,433) |

*CI – confidence interval; PTSD – post-traumatic stress disorder*
