# Supplementary material for: Illicit Cannabis Use to Self-Treat Chronic Health Conditions in the United Kingdom: Cross-Sectional Study
Source: JMIR Public Health Surveill. 2024 Aug 14;10:e57595. doi: 10.2196/57595 (PMC11337234; doi:10.2196/57595)
Supplement: Multimedia Appendix 2 [file publichealth-v10-e57595-s002.docx]

|  | Chronic Pain | Anxiety | Fibro-myalgia | PTSD | Multiple Sclerosis | Other mental health condition | Other physical condition | Other, not described | Any Condition |
| --- | --- | --- | --- | --- | --- | --- | --- | --- | --- |
| **Gender** | | | | | | | | | |
| **Male** | 353 (42.96%) | 603  (37.98%) | 73  (23.96%) | 161 (39.77%) | 49  (47.14%) | 724  (44.88%) | 1,231  (45.68%) | 550  (51.70%) | 2,597  (45.57%) |
| **Female** | 468 (57.04%) | 985  (62.02%) | 232  (76.04%) | 244  (60.23%) | 55  (52.86%) | 889  (55.12%) | 1,464  (54.33%) | 514  (48.30%) | 3,072  (53.90%) |
| **Age** | | | | | | | | | |
| **18-24** | 58  (7.01%) | 271  (17.09%) | 42  (13.76%) | 71  (17.55%) | 22  (21.10%) | 255  (15.80%) | 138  (5.11%) | 46  (4.31%) | 563  (9.88%) |
| **25-34** | 77  (9.43%) | 365  (23.01%) | 38  (12.34%) | 74  (18.18%) | 21  (20.33%) | 313  (19.39%) | 223  (8.29%) | 82  (7.70%) | 712  (12.49%) |
| **35-44** | 111  (13.52%) | 356  (22.43%) | 45  (14.79%) | 84  (20.71%) | 28  (27.24%) | 381  (23.61%) | 318  (11.81%) | 141  (13.21%) | 900  (15.78%) |
| **45-54** | 162  (19.75%) | 254  (15.99%) | 55  (17.93%) | 70  (17.22%) | 10  (9.31%) | 288  (17.85%) | 411  (15.25%) | 150  (14.12%) | 840  (14.74%) |
| **55+** | 413  (50.30%) | 341  (21.47%) | 126  (41.18%) | 107  (26.34%) | 23  (22.02%) | 377  (23.34%) | 1,605  (59.55%) | 646  (60.67%) | 2,656  (46.59%) |
| **Social Class** | | | | | | | | | |
| **ABC1** | 358  (43.61%) | 756  (47.60%) | 145  (47.49%) | 187 (46.17%) | 69  (66.82%) | 779 (48.30%) | 1,495  (55.46%) | 580  (54.44%) | 3,112  (54.60%) |
| **C2DE** | 463  (56.39%) | 832  (52.40%) | 160  (52.51%) | 218  (53.83%) | 34  (33.18%) | 834  (51.70%) | 1,200  (44.54%) | 485  (45.56%) | 2,558  (44.87%) |
| **Region** | | | | | | | | | |
| **East Midlands** | 62  (7.61%) | 104  (6.54%) | 34  (11.13%) | 26  (6.29%) | 6  (5.51%) | 91  (5.62%) | 220  (8.17%) | 67  (6.28%) | 405  (7.10%) |
| **East of England** | 57  (6.91%) | 122  (7.69%) | 27  (8.97%) | 28  6.83%) | 11  (10.24%) | 135  (8.39%) | 219  (8.12%) | 98  (9.21%) | 488  (8.56%) |
| **London** | 90  (10.94%) | 176  (11.07%) | 36  (11.76%) | 56  (13.90%) | 20  (19.44%) | 187  (11.59%) | 322  (11.96%) | 125  (11.77%) | 684  (12.01%) |
| **North East** | 36  (4.40%) | 72  (4.54%) | 14  (4.70%) | 19  (4.68%) | 3  (2.88%) | 80  (4.93%) | 96  (3.56%) | 39  (3.71%) | 233  (4.10%) |
| **North West** | 87  (10.56%) | 183  (11.53%) | 33  (10.87%) | 31  (7.52%) | 8  (8.14%) | 170  (10.53%) | 283  (10.49%) | 137  (12.87%) | 611  (10.73%) |
| **Northern Ireland** | 35  (4.21%) | 33  (2.07%) | 11  (3.64%) | 17  (4.13%) | 2  (1.88%) | 57  (3.54%) | 74  (2.76%) | 28  (2.60%) | 157  (2.75%) |
| **Scotland** | 98  (11.98%) | 132  (8.29%) | 30  (9.71%) | 38  (9.35%) | 6  (5.59%) | 139  (8.60%) | 245  (9.11%) | 83  (7.78%) | 479  (8.41%) |
| **South East** | 96  (11.65%) | 212  (13.34%) | 31  (10.00%) | 59  (14.49%) | 11  (10.49%) | 213  (13.20%) | 355  (13.18%) | 165  (15.52%) | 767  (13.46%) |
| **South West** | 76  (9.29%) | 144  (9.09%) | 21  (6.72%) | 45  (11.15%) | 8  (7.59%) | 164  (10.17%) | 288  (10.69%) | 112  (10.47%) | 561  (9.84%) |
| **Wales** | 49  (6.03%) | 105  (6.60%) | 17  (5.57%) | 27  (6.68%) | 6  (5.63%) | 104  (6.46%) | 136  (5.04%) | 46  (4.33%) | 289  (5.07%) |
| **West Midlands** | 67  (8.15%) | 165  (10.37%) | 32  (10.35%) | 27  (6.60%) | 9  (8.97%) | 114  (7.05%) | 234  (8.66%) | 75  (7.02%) | 489  (8.58%) |
| **Yorkshire and the Humber** | 68  (8.28%) | 141  (8.88%) | 20  (6.58%) | 34  (8.39%) | 14  (13.62%) | 160  (9.93%) | 223  (8.26%) | 90  (8.44%) | 506  (8.87%) |
| **Country** | | | | | | | | | |
| **England** | 638  (77.78%) | 1,318  (83.04%) | 248  (81.08%) | 324  (79.84%) | 90  (86.69%) | 1,313  (81.41%) | 2,240  (83.10%) | 908  (85.29%) | 4,744  (83.24%) |
| **Northern Ireland** | 35  (4.21%) | 33  (2.07%) | 11  (3.64%) | 17  (4.13%) | 2  (1.88%) | 57  (3.54%) | 74  (2.76%) | 28  (2.60%) | 157  (2.75%) |
| **Scotland** | 98  (11.98%) | 132  (8.29%) | 30  (9.71%) | 38  (9.35%) | 6  (5.59%) | 139  (8.60%) | 245  (9.11%) | 83  (7.78%) | 479  (8.41%) |
| **Wales** | 49  (6.03%) | 105  (6.60%) | 17  (5.57%) | 27  (6.68%) | 6  (5.63%) | 104  (6.46%) | 136  (5.04%) | 46  (4.33%) | 289  (5.07%) |
| **Employment** | | | | | | | | | |
| **Full time student** | 28  (3.38%) | 125  (7.85%) | 17  (5.64%) | 25  (6.18%) | 9  (9.07%) | 112  (6.95%) | 70  (2.61%) | 26  (2.46%) | 248  (4.35%) |
| **Not working, Other** | 211  (25.68%) | 313  (19.69%) | 95  (30.95%) | 91  (22.49%) | 17  (16.48%) | 331  (20.51%) | 349  (12.96%) | 94  (8.85%) | 703  (12.33%) |
| **Retired** | 264  (32.15%) | 173  (10.89%) | 67  (22.07%) | 64  (15.67%) | 14  (13.57%) | 177  (10.95%) | 1,046  (38.82%) | 408  (38.36%) | 1,659  (29.10%) |
| **Unemployed** | 42  (5.08%) | 142  (8.95%) | 13  (4.27%) | 32  (7.87%) | 6  (5.76%) | 139  (8.64%) | 96  (3.57%) | 41  (3.85%) | 286  (5.02%) |
| **Working full time** | 167  (20.33%) | 605  (38.12%) | 60  (19.51%) | 131  (32.32%) | 39  (37.54%) | 638  (39.56%) | 787  (29.21%) | 350  (32.89%) | 1,975  (34.66%) |
| **Working part time** | 110  (13.38%) | 230  (14.50%) | 54  (17.56%) | 63  (15.47%) | 18  (17.57%) | 216  (13.39%) | 346  (12.83%) | 145  (13.59%) | 799  (14.01%) |
| **Marital Status** | | | | | | | | | |
| **Living as married** | 97  (11.78%) | 253  (15.92%) | 34  (11.17%) | 51  (12.53%) | 15  (14.01%) | 221  (13.69%) | 314  (11.65%) | 123  (11.55%) | 718  (12.60%) |
| **Married/ Civil Partnership** | 374  (45.62%) | 479  (30.17%) | 139 (45.62%) | 129  (31.76%) | 45  (43.12%) | 506  (31.34%) | 1,318  (48.89%) | 538  (50.51%) | 2,518  (44.17%) |
| **Never Married** | 208  (25.29%) | 680  (42.85%) | 74  (24.16%) | 166  (40.90%) | 32  (30.73%) | 698  (43.27%) | 600  (22.27%) | 219  (20.58%) | 1,593  (27.95%) |
| **Separated/ Divorced** | 101  (12.26%) | 137  (8.63%) | 41  (13.37%) | 50  (12.21%) | 8  (7.43%) | 155  (9.63%) | 301  (11.18%) | 113  (10.57%) | 565  (9.91%) |
| **Widowed** | 40  (4.92%) | 36  (2.29%) | 17  (5.68%) | 10  (2.37%) | 5  (4.72%) | 29  (1.83%) | 160  (5.94%) | 69  (6.45%) | 267  (4.68%) |
| **Number of Children in Household** | | | | | | | | | |
| **No Children** | 646  (78.68%) | 1,141  (71.86%) | 209  (68.55%) | 284  (69.92%) | 53  (51.50%) | 1,186  (73.56%) | 2,234  (82.88%) | 881  (82.72%) | 4,396  (77.12%) |
| **1 Child** | 92  (11.16%) | 221  (13.89%) | 43  (14.12%) | 60  (14.89%) | 12  (11.85%) | 223  (13.85%) | 213  (7.91%) | 92  (8.60%) | 594  (10.43%) |
| **2 Children** | 49  (6.00%) | 146  (9.17%) | 29  (9.53%) | 37  (9.12%) | 22  (21.66%) | 137  (8.51%) | 187  (6.93%) | 69  (6.49%) | 469  (8.22%) |
| **3+ Children** | 34  (4.15%) | 81  (5.08%) | 24  (7.79%) | 25  (6.07%) | 16  (14.99%) | 66  (4.08%) | 61  (2.28%) | 23  (2.19%) | 211  (3.70%) |

*ABC1 – middle class; C2DE – working class or not working*
