# Supplementary material for: Illicit Cannabis Use to Self-Treat Chronic Health Conditions in the United Kingdom: Cross-Sectional Study
Source: JMIR Public Health Surveill. 2024 Aug 14;10:e57595. doi: 10.2196/57595 (PMC11337234; doi:10.2196/57595)
Supplement: Multimedia Appendix 3 [file publichealth-v10-e57595-s003.docx]

|  | Chronic Pain | Anxiety | Fibro-myalgia | PTSD | Multiple Sclerosis | Other mental health condition | Other physical condition | Other, not described | Any Condition |
| --- | --- | --- | --- | --- | --- | --- | --- | --- | --- |
| **Gender** | | | | | | | | | |
| **Male** | 47  (50.55%) | 76  (47.99%) | 24  (52.04%) | 42  (56.08%) | 25  (62.54%) | 87  (49.44%) | 65  (51.10%) | 13  (48.30%) | 201  (55.20%) |
| **Female** | 46  (49.45%) | 83  (52.01%) | 22  (47.96%) | 33  (43.92%) | 15  (37.46%) | 89  (50.56%) | 62  (48.90%) | 14  (51.70%) | 163  (44.80%) |
| **Age** | | | | | | | | | |
| **18-24** | 16  (17.35%) | 39  (24.61%) | 18  (38.83%) | 27  (36.31%) | 11  (28.66%) | 47  (26.80%) | 21  (16.45%) | 7  (24.11%) | 106  (29.15%) |
| **25-34** | 18  (19.43%) | 51  (31.89%) | 11  (23.36%) | 19  (25.51%) | 11  (26.71%) | 40  (22.59%) | 17  (13.78%) | 4  (14.57%) | 86  (23.77%) |
| **35-44** | 22  (23.34%) | 44  (27.69%) | 13  (28.14%) | 16  (21.93%) | 13  (32.30%) | 50  (28.35%) | 33  (26.13%) | 8  (30.61%) | 86  (23.61%) |
| **45-54** | 17  (18.33%) | 19  (11.98%) | 1  (2.12%) | 8  (10.88%) | 3  (7.16%) | 27  (15.20%) | 27  (21.09%) | 4  (14.14%) | 45  (12.47%) |
| **55+** | 20  (21.55%) | 6  (3.83%) | 4  (7.56%) | 4  (5.37%) | 2  (5.16%) | 12  (7.06%) | 29  (22.55%) | 5  (16.58%) | 40  (11.00%) |
| **Social Class** | | | | | | | | | |
| **ABC1** | 43  (45.76%) | 66  (41.62%) | 32  (68.95%) | 34  (45.60%) | 26  (65.62%) | 70  (39.69%) | 46  (36.08%) | 15  (52.71%) | 176  (48.39%) |
| **C2DE** | 51  (54.23%) | 93  (58.38%) | 15  (31.05%) | 41  (54.40%) | 14  (34.38%) | 106  (60.31%) | 81  (63.92%) | 13  (47.29%) | 188  (51.61%) |
| **Region** | | | | | | | | | |
| **East Midlands** | 3  (2.76%) | 5  (3.26%) | 8  (17.93%) | 1  (1.30%) | 2  (5.11%) | 11  (6.24%) | 7  (5.40%) | 1  (3.72%) | 19  (5.32%) |
| **East of England** | 10  (10.44%) | 11  (6.69%) | 4  (7.92%) | 2  (2.36%) | 1  (2.48%) | 8  (4.61%) | 6  (4.58%) | 1  (3.09%) | 23  (6.45%) |
| **London** | 13  (14.24%) | 29  (18.19%) | 15  (32.44%) | 18  (24.37%) | 14  (34.65%) | 30  (17.22%) | 16  (12.44%) | 5  (16.67%) | 74  (20.25%) |
| **North East** | 6  (5.96%) | 6  (3.52%) | 0  (0.00%) | 5  (7.31%) | 0  (0.00%) | 8  (4.27%) | 8  (6.33%) | 0  (0.00%) | 15  (4.11%) |
| **North West** | 11  (11.88%) | 22  (13.66%) | 5  (10.31%) | 7  (9.31%) | 4  (9.08%) | 23  (13.16%) | 19  (14.85%) | 6  (20.21%) | 43  (11.88%) |
| **Northern Ireland** | 7  (7.20%) | 3  (1.81%) | 2  (4.59%) | 0  (0.00%) | 1  (2.59%) | 12  (6.70%) | 6  (4.67%) | 0  (0.00%) | 17  (4.81%) |
| **Scotland** | 10  (10.94%) | 12  (7.47%) | 3  (5.38%) | 5  (7.26%) | 2  (4.84%) | 11  (6.25%) | 14  (11.13%) | 5  (17.26%) | 28  (7.72%) |
| **South East** | 12  (13.13%) | 20  (12.62%) | 6  (12.83%) | 11  (14.77%) | 2  (5.32%) | 19  (10.85%) | 17  (13.51%) | 6  (20.38%) | 39  (10.61%) |
| **South West** | 7  (7.28%) | 15  (9.29%) | 0  (0.00%) | 7  (9.55%) | 1  (2.34%) | 15  (8.55%) | 13  (9.92%) | 4  (15.07%) | 28  (7.62%) |
| **Wales** | 4  (3.82%) | 12  (7.36%) | 0  (0.00%) | 4  (5.35%) | 5  (12.30%) | 12  (6.70%) | 8  (6.17%) | 1  (3.60%) | 22  (6.16%) |
| **West Midlands** | 5  (4.85%) | 16  (10.02%) | 0  (0.00%) | 4  (5.35%) | 4  (8.95%) | 9  (5.21%) | 8  (6.22%) | 0  (0.00%) | 24  (6.66%) |
| **Yorkshire and the Humber** | 7  (7.51%) | 10  (6.11%) | 4  (8.59%) | 10  (13.09%) | 5  (12.34%) | 18  (10.25%) | 6  (4.78%) | 0  (0.00%) | 31  (8.41%) |
| **Country** | | | | | | | | | |
| **England** | 73  (78.05%) | 133  (83.36%) | 42  (90.02%) | 66  (87.39%) | 32  (80.27%) | 142  (80.35%) | 99  (78.03%) | 22  (79.14%) | 296  (81.31%) |
| **Northern Ireland** | 7  (7.20%) | 3  (1.81%) | 2  (4.59%) | 0  (0.00%) | 1  (2.59%) | 12  (6.70%) | 6  (4.67%) | 0  (0.00%) | 17  (4.81%) |
| **Scotland** | 10  (10.94%) | 12  (7.47%) | 3  (5.38%) | 5  (7.26%) | 2  (4.84%) | 11  (6.25%) | 14  (11.13%) | 5  (17.26%) | 28  (7.72%) |
| **Wales** | 4  (3.82%) | 12  (7.36%) | 0  (0.00%) | 4  (5.35%) | 5  (12.30%) | 12  (6.70%) | 8  (6.17%) | 1  (3.60%) | 22  (6.16%) |
| **Employment** | | | | | | | | | |
| **Full time student** | 4  (4.10%) | 17  (10.44%) | 9  (18.30%) | 3  (4.08%) | 5  (13.10%) | 16  (8.98%) | 9  (7.03%) | 5  (18.66%) | 37  (10.25%) |
| **Not working, Other** | 28  (30.52%) | 42  (26.32%) | 5  (10.97%) | 16  (21.75%) | 5  (12.93%) | 51  (28.68%) | 34  (26.84%) | 10  (35.30%) | 78  (21.46%) |
| **Retired** | 12  (12.98%) | 3  (1.97%) | 3  (5.62%) | 4  (4.98%) | 1  (2.59%) | 6  (3.64%) | 15  (11.58%) | 3  (9.79%) | 24  (6.66%) |
| **Unemployed** | 8  (8.10%) | 22  (13.51%) | 6  (13.01%) | 11  (14.95%) | 5  (12.28%) | 29  (16.63%) | 13  (10.25%) | 4  (13.70%) | 49  (13.42%0 |
| **Working full time** | 24  (25.84%) | 43  (27.01%) | 13  (27.05%) | 23  (31.24%) | 17  (42.12%) | 50  (28.41%) | 40  (31.58%) | 4  (15.88%) | 111  (30.64%) |
| **Working part time** | 17  (18.46%) | 33  (20.75%) | 12  (25.05%) | 17  (23.01%) | 7  (16.97%) | 24  (13.66%) | 16  (12.71%) | 2  (6.67%) | 64  (17.56%) |
| **Marital Status** | | | | | | | | | |
| **Living as married** | 15  (16.07%) | 21  (13.03%) | 4  (7.79%) | 3  (4.32%) | 5  (12.72%) | 24  (13.56%) | 21  (16.35%) | 5  (17.75%) | 48  (13.10%) |
| **Married/ Civil Partnership** | 27  (28.97%) | 38  (23.58%) | 19  (40.47%) | 21  (27.56%) | 17  (42.38%) | 32  (18.24%) | 34  (27.13%) | 6  (20.52%) | 93  (25.69%) |
| **Never Married** | 35  (37.39%) | 94  (29.03%) | 18  (37.43%) | 45  (60.28%) | 13  (32.30%) | 106  (60.01%) | 50  (39.41%) | 15  (55.36%) | 186  (51.05%) |
| **Separated/ Divorced** | 14  (15.35%) | 6  (3.64%) | 3  (6.10%) | 5  (6.32%) | 3  (7.24%) | 12  (7.01%) | 20  (15.47%) | 1  (3.27%) | 30  (8.34%) |
| **Widowed** | 2  (2.22%) | 1  (0.72%) | 4  (8.20%) | 1  (1.52%) | 2  (5.36%) | 2  (1.17%) | 2  (1.65%) | 1  (3.09%) | 7  (1.82%) |
| **Number of Children in Household** | | | | | | | | | |
| **No Children** | 70  (75.08%) | 103  (64.86%) | 24  (51.60%) | 51  (68.51%) | 16  (39.59%) | 131  (74.10%) | 96  (75.57%) | 17  (61.26%) | 237  (65.28%) |
| **1 Child** | 14  (14.80%) | 19  (12.21%) | 7  (14.93%) | 12  (15.70%) | 6  (14.04%) | 21  (11.96%) | 14  (11.11%) | 4  (14.53%) | 48  (13.13%) |
| **2 Children** | 2  (2.05%) | 15  (9.46%) | 8  (17.51%) | 7  (9.25%) | 10  (24.55%) | 12  (6.88%) | 7  (5.55%) | 5  (17.35%) | 38  (10.57%) |
| **3+ Children** | 8  (8.08%) | 21  (13.47%) | 7  (15.96%) | 5  (6.54%) | 9  (21.82%) | 12  (7.06%) | 10  (7.78%) | 2  (6.85%) | 40  (11.03%) |

*ABC1 – middle class; C2DE – working class or not working.*
