# Supplementary material for: Illicit Cannabis Use to Self-Treat Chronic Health Conditions in the United Kingdom: Cross-Sectional Study
Source: JMIR Public Health Surveill. 2024 Aug 14;10:e57595. doi: 10.2196/57595 (PMC11337234; doi:10.2196/57595)
Supplement: Multimedia Appendix 4 [file publichealth-v10-e57595-s004.docx]

| Condition | £1 to £99 | £100 to £199 | £200 to £299 | £300 to £399 | £400 or above | Don't know | Not applicable - I do not spend any money | Prefer not to say |
| --- | --- | --- | --- | --- | --- | --- | --- | --- |
| Chronic Pain | 35  (38.07%) | 17  (17.98%) | 14  (15.28%) | 7  (7.24%) | 4  (4.29%) | 2  (2.13%) | 6  (6.51%) | 8  (8.50%) |
| Anxiety | 67  (42.06%) | 34  (21.40%) | 20  (12.55%) | 12  (7.74%) | 6  (3.96%) | 4  (2.67%) | 5  (3.38%) | 10  (6.24%) |
| Fibromyalgia | 10  (21.40%) | 9  (19.45%) | 6  (12.88%) | 11  (23.99%) | 6  (13.04%) | 1  (1.95%) | 1  (1.93%) | 3  (5.36%) |
| PTSD | 30  (39.49%) | 17  (22.76%) | 10  (12.71%) | 4  (4.82%) | 6  (7.57%) | 0  (0.00%) | 5  (7.00%) | 4  (5.65%) |
| Multiple Sclerosis | 6  (15.81%) | 8  (20.59%) | 5  (13.73%) | 12  (29.77%) | 6  (15.41%) | 2  (4.68%) | 0  (0.00%) | 0  (0.00%) |
| Mental Health | 79  (44.57%) | 39  (22.38%) | 17  (9.39%) | 14  (8.13%) | 4  (2.54%) | 3  (1.77%) | 11  (6.21%) | 9  (5.01%) |
| Physical Condition | 61  (48.34%) | 25  (19.42%) | 16  (12.66%) | 3  (2.52%) | 4  (3.39%) | 2  (1.59%) | 7  (5.42%) | 8  (6.66%) |
| Other Conditions | 11  (38.31%) | 3  (11.01%) | 1  (3.56%) | 2  (8.81%) | 0  (0.00%) | 1  (3.30%) | 6  (21.49%) | 4  (13.53%) |
| **Any Condition** | 134  (36.85%) | 68  (18.69%) | 47  (12.85%) | 36  (9.88%) | 24  (6.59%) | 12  (3.37%) | 24  (6.58%) | 19  (5.19%) |

*PTSD – post-traumatic stress disorder*
