# Supplementary material for: Illicit Cannabis Use to Self-Treat Chronic Health Conditions in the United Kingdom: Cross-Sectional Study
Source: JMIR Public Health Surveill. 2024 Aug 14;10:e57595. doi: 10.2196/57595 (PMC11337234; doi:10.2196/57595)
Supplement: Multimedia Appendix 5 [file publichealth-v10-e57595-s005.docx]

| Conditions | Unaware medical cannabis was available legally to manage / treat some conditions | Presumed obtaining it legally would be very difficult | Presumed obtaining it legally would be very expensive | Wanted to treat condition quickly | Other | Don't know | Prefer not to say |
| --- | --- | --- | --- | --- | --- | --- | --- |
| Chronic Pain | 26  (27.83%) | 34  (36.72%) | 31  (33.04%) | 34  (36.21%) | 9  (9.13%) | 3  (2.82%) | 3  (3.07%) |
| Anxiety | 37  (23.48%) | 80  (50.10%) | 47  (29.64%) | 54  (34.08%) | 13  (8.36%) | 6  (3.64%) | 2  (1.35%) |
| Fibromyalgia | 10  (22.29%) | 20  (43.38%) | 14  (29.63%) | 16  (34.69%) | 5  (10.83%) | 4  (8.81%) | 0  (0.00%) |
| PTSD | 17  (23.29%) | 33  (43.86%) | 30  (40.27%) | 33  (44.46%) | 5  (6.46%) | 0  (0.00%) | 2  (2.80%) |
| Multiple Sclerosis | 4  (10.77%) | 17  (42.94%) | 18  (45.77%) | 11  (27.48%) | 0  (0.00%) | 2  (5.64%) | 0  (0.00%) |
| Mental Health | 54  (30.42%) | 66  (37.55%) | 51  (29.15%) | 53  (30.12%) | 17  (9.87%) | 5  (2.76%) | 8  (4.72%) |
| Physical Condition | 27  (21.58%) | 61  (48.58%) | 40  (31.33%) | 48  (37.57%) | 16  (12.40%) | 1  (0.74%) | 5  (3.91%) |
| Other Conditions | 2  (7.75%) | 17  (61.20%) | 6  (23.40%) | 10  (37.09%) | 6  (20.52%) | 3  (10.25%) | 1  (3.09%) |
| **Any Condition** | 88  (24.15%) | 148  (40.75%) | 105  (28.87%) | 103  (28.35%) | 29  (7.86%) | 16  (4.26%) | 12  (3.29%) |

*PTSD – post-traumatic stress disorder*
