# Supplementary material for: Illicit Cannabis Use to Self-Treat Chronic Health Conditions in the United Kingdom: Cross-Sectional Study
Source: JMIR Public Health Surveill. 2024 Aug 14;10:e57595. doi: 10.2196/57595 (PMC11337234; doi:10.2196/57595)
Supplement: Multimedia Appendix 6 [file publichealth-v10-e57595-s006.docx]

| Conditions | Don't know/can't recall | I mentioned it but my GP/ specialist advised against it | I mentioned it to my GP/ specialist but they knew nothing about it | My GP/ specialist mentioned it in passing | None of these | Prefer not to say | We have discussed it in detail and decided against it | We have discussed it in detail and explored it/ are exploring it further | We have never discussed it |
| --- | --- | --- | --- | --- | --- | --- | --- | --- | --- |
| Chronic Pain | 3  (3.01%) | 6  (6.86%) | 10  (11.21%) | 11  (12.12%) | 3  (2.82%) | 1  (1.10%) | 3  (3.12%) | 11  (11.46%) | 45  (48.30%) |
| Anxiety | 2  (1.30%) | 14  (9.06%) | 24  (14.81%) | 3  (1.72%) | 10  (6.17%) | 1  (0.64%) | 4  (2.43%) | 12  (7.68%) | 90  (56.19%) |
| Fibromyalgia | 2  (4.40%) | 5  (10.81%) | 10  (21.11%) | 11  (22.73%) | 1  (1.79%) | 0  (0.00%) | 5  (10.55%) | 5  (10.44%) | 8  (18.15%) |
| PTSD | 3  (4.10%) | 10  (12.81%) | 10  (13.02%) | 6  (7.79%) | 4  (5.05%) | 0  (0.00%) | 3  (3.83%) | 15  (19.48%) | 25  (33.93%) |
| Multiple Sclerosis | 0  (0.00%) | 7  (16.91%) | 9  (22.68%) | 5  (12.24%) | 3  (7.94%) | 0  (0.00%) | 6  (15.36%) | 7  (17.62%) | 3  (7.26%) |
| Mental Health | 6  (3.58%) | 8  (4.53%) | 19  (10.51%) | 7  (4.11%) | 10  (5.54%) | 2  (1.12%) | 6  (3.60%) | 14  (8.11%) | 104  (58.90%) |
| Physical Condition | 2  (1.40%) | 8  (6.64%) | 11  (8.69%) | 3  (2.34%) | 12  (9.41%) | 4  (3.17%) | 0  (0.00%) | 11  (8.45%) | 76  (59.89%) |
| Other Conditions | 3  (10.67%) | 1  (3.51%) | 0  (0.00%) | 2  (6.63%) | 2  (7.11%) | 0  (0.00%) | 1  (3.30%) | 3  (11.16%) | 16  (57.62%) |
| **Any Condition** | 10  (2.78%) | 31  (8.40%) | 43  (11.86%) | 23  (6.27%) | 23  (6.30%) | 5  (1.38%) | 19  (5.18%) | 35  (9.71%) | 175  (48.11%) |

*GP – general practitioner; PTSD – post-traumatic stress disorder*
